# Efficacy and safety of acupuncture on relieving abdominal pain and distension for acute pancreatitis: A protocol for systematic review

**DOI:** 10.1097/MD.0000000000019044

**Published:** 2020-02-21

**Authors:** Xinyun Zhu, Lijie Yang, Xianglei Li, Fengya Zhu, Zimeng Li, Andrea Craemer, Yueheng Xiong, Ying Lan, Yuemeng Zhao, Jie Wu

**Affiliations:** a Acupuncture and Moxibustion School, Chengdu University of Traditional Chinese Medicine; b Hospital of Chengdu University of Traditional Chinese Medicine, Jinniu District, Chengdu, Sichuan; c The Second Affiliated Hospital of Xingtai Medical College, Qiaoxi District, Xingtai, Hebei; d School of Basic Medical Science, Chengdu University of Traditional Chinese Medicine, Jinniu District, Chengdu, Sichuan, China.

**Keywords:** acupuncture, acute pancreatitis, protocol, systematic review

## Abstract

**Introduction:**

The purpose of this study is to evaluate the efficacy and safety of acupuncture on relieving abdominal pain and distension in acute pancreatitis.

**Methods and analysis:**

We will electronically search PubMed, MEDLINE, Embase, Web of Science, the Cochrane Central Register of Controlled Trial, China National Knowledge Infrastructure, China Biomedical Literature Database, China Science Journal Database, and Wanfang Database from their inception. Furthermore, we will manually retrieve other resources, including reference lists of identified publications, conference articles, and gray literature. The clinical randomized controlled trials or quasi-randomized controlled trials related to acupuncture treating acute pancreatitis will be included in the study. The language is limited to Chinese and English. Research selection, data extraction, and research quality assessment will be independently completed by 2 researchers. Data will be synthesized using a fixed effects model or random effects model depending on the heterogeneity test. The overall response rate and the visual analog scale score will be the primary outcomes. The time of first bowel sound, the time of first defecation, the length of hospitalization, acute physiology and chronic health evaluation II score, and the adverse events will also be assessed as secondary outcomes. RevMan 5 (version 5.3) statistical software will be used for meta-analysis, and the level of evidence will be assessed by Grading of Recommendations Assessment, Development, and Evaluation. Continuous data will be expressed in the form of weighted mean difference or standardized mean difference with 95% confidence intervals, whereas dichotomous data will be expressed in the form of risk ratios with 95% confidence intervals.

**Ethics and dissemination:**

The protocol of this systematic review does not require ethical approval because it does not involve humans. We will publish this article in peer-reviewed journals and present at relevant conferences.

**PROSPERO registration number:**

CRD42019147503.

## 1 Introduction

Acute pancreatitis (AP) is an inflammatory disorder of the pancreas, which is the leading cause of hospitalization among gastrointestinal disorders in many countries.^[1]^ Its annual incidence was reported as 13 to 45 cases per 100,000 persons, and it is associated with mortality up to 30% in severe cases.^[2,3]^ The routine treatment of AP includes fluid resuscitation, nutrition, antibiotics as well as advanced endoscopic techniques, open surgery drainage, or cholecystectomy for severe or specific types.^[4]^

On AP patients, upper abdominal pain is a major symptom, which is mostly treated by opioids, but increased opioid use was found to be associated with longer hospitalization, suggesting improved approaches to pain control for AP patients are necessary.^[5]^ Besides, at the initial stage of AP, bowel dysfunction, manifesting as abdominal distension, is common and a risk factor for toxic and inflammatory products accumulation, which may induce systemic inflammatory response syndrome and multiple organ dysfunction syndrome.^[6–8]^

Acupuncture is an important form of traditional Chinese medicine and its analgesic function is widely proven.^[9]^ Studies have reported that acupuncture can effectively relieve abdominal pain and facilitate the gastrointestinal function in AP patients,^[10,11]^ but the quality of these evidences remains low. Thus, this systematic review (SR) aims to assess the efficacy and safety of acupuncture in relieving abdominal pain and distension in AP.

## 2 Methods

The protocol has been registered on PROSPERO as CRD42019147503 (https://www.crd.york.ac.uk/PROSPERO/display_record.php?RecordID=147503). The protocol follows the Preferred Reporting Items for Systematic Reviews and Meta-Analyses Protocols 2015 statement guidelines.^[12]^ We will report the changes in the full review if necessary.

### 2.1 Inclusion and exclusion criteria for study selection

#### 2.1.1 Inclusion criteria

This study will include randomized controlled trials (RCTs) of acupuncture as additional treatment of AP, whether using blind method or allocation concealment method. The language of the trials to be included should be Chinese or English.

#### 2.1.2 Exclusion criteria

Following studies would be excluded:

1. patient age < 18 years;
2. pregnancy;
3. case reports and reviews;
4. literature not in English or Chinese language;
5. clinical research studies that compared different kinds of acupuncture or moxibustion;
6. the treatment was combined with other treatments other than acupuncture; and
7. animal studies.

### 2.2 Types of participants

Types of participants included patients diagnosed with AP, both mild and severe type, pain visual analog scale (VAS) score ≥ 4, with no limitation to gender. All of participants should be hospitalized and accept acupuncture treatment. Patients under 18 years of age or pregnant patients would be excluded.

### 2.3 Types of interventions

The acupuncture treatment should be an additional treatment on the basis of routine treatments, including traditional acupuncture, electroacupuncture, laser acupuncture, auricular acupuncture, etc; the details of acupuncture treatment should be clearly illustrated according to STRICTRA, involving the needle selection, acupoints selection, manipulations, course, etc.^[13]^

### 2.4 Control

The control interventions include routine treatments alone or routine treatments plus sham/placebo acupuncture. Meanwhile, the control group should receive same routine treatment as acupuncture group.

The following studies would be excluded:

1. RCTs that compare different kinds of acupuncture;
2. placebo-controlled or waiting list control group; and
3. acupuncture group and control group received different routine treatment.

### 2.5 Types of outcome measures

#### 2.5.1 Primary outcomes

We use the following outcomes:

1. the overall response rate;
2. the VAS scores of abdominal pain and distension; and
3. the time of pain and distension relieve.

#### 2.5.2 Secondary outcomes

We also care about the following indexes:

1. the time of first bowel sound;
2. the time of first defecation;
3. the length of hospitalization;
4. acute physiology and chronic health evaluation II (APACHE II) score; and
5. adverse events.

## 3 Data sources

### 3.1 Electronic searches

Following databases will be searched: PubMed, Web of Science, the Cochrane Central Register of Controlled Trials, AMED, MEDLINE, Embase, Cochrane Library, China National Knowledge Infrastructure, Wanfang data, Chinese Scientific Journals Database (VIP), and China Biomedical Literature Database. We selected the eligible studies published up to November 30, 2019. The search terms used in the SR are as follows: acupuncture, acute pancreatitis, pancreatitis, budemian, and budeming. We will not apply any language, population, or national restrictions. The specific search strategy (taking PubMed as an example) is listed on Table 1. Similar search strategy will be applied to other electronic databases.

**Table 1:**
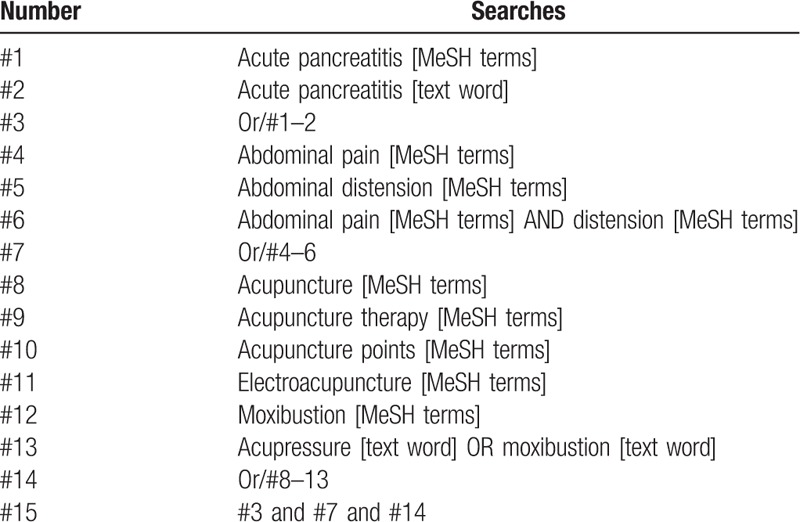
Search strategy sample of PubMed.

We will identify relevant RCTs and the selected studies will be analyzed according to the Cochrane Handbook.

### 3.2 Searching other resources

**Figure 1:**
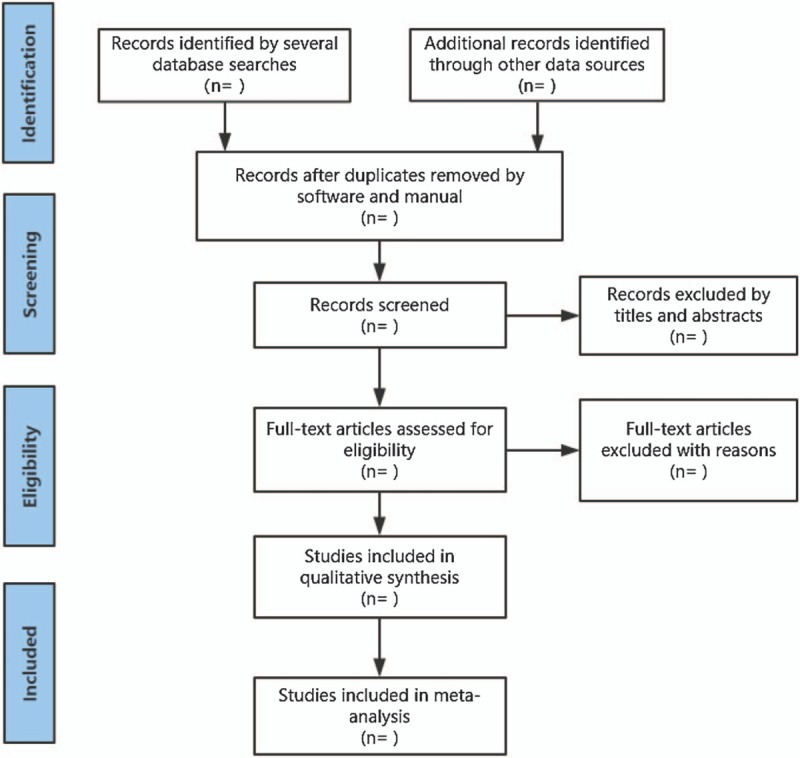
The research flowchart.

We will also retrieve manual-related documents, such as replacing and supplementing some reference documents, medical textbooks, clinical laboratory manuals, and the World Health Organization International Clinical Trials Registry Platform. At the same time, we will contact experts and authors in this field to obtain important information that cannot be found in the search. The research flowchart is shown in Figure 1.

## 4 Data collection and analysis

### 4.1 Selection of studies

Two independent researchers (ZX and ZY) will assess the full-text articles from the search results independently against the inclusion and exclusion criteria. Discrepancies will be discussed and resolved by consensus with a third author (LX).

### 4.2 Data extraction and management

The following information will be extracted from each study: research number, data extractor, date of data extraction, general situation of the study, research methodology, research population, baseline comparability, interventions, main outcome indicators, secondary outcome indicators, combined drug use, adverse reactions or complications, etc. For those with questions or incomplete information, we will try to contact the author to obtain information before deciding whether to include it.

### 4.3 Assessment of the reporting quality and risk of bias

Two of the authors (ZX and ZY) individually assessed the risk of bias using assessments included in the study were evaluated in the Cochrane System Evaluator's Manual for RCT quality evaluation criteria. Assessing the risk of bias:

1. random sequence generation;
2. allocation concealment;
3. blinding of participants and personnel;
4. blinding of outcome assessment;
5. incomplete outcome data;
6. selective outcome reporting; and
7. other bias.

Every domain was classified as high risk of bias, low risk of bias, or unclear risk of bias. Any arising difference was resolved by discussion.

### 4.4 Measures of a treatment effect

We will measure continuous data with mean difference or standard mean difference for the therapeutic effect with 95% confidence intervals. For dichotomous data, risk ratios with 95% confidence intervals will be calculated.

### 4.5 Management of missing data

To obtain the missing data, we will contact the corresponding author. If no response will be obtained, we will analyze only the available data and describe the reason and impact of this exclusion in the paper.

### 4.6 Assessment of a reporting bias

Publication bias will be explored through funnel plot analysis. Grading of Recommendations Assessment, Development, and Evaluation (GRADE) profiler 3.6 is used to evaluate the quality of evidence. The specific contents include limitations of research, inconsistency of research results, indirect evidence, inadequate accuracy, and publication bias. Finally, the quality of evidence is divided into 4 levels: high-level evidence, intermediate evidence, low-level evidence, and very low-level evidence.

### 4.7 Assessment of heterogeneity

All literatures will use *I*^2^ value of the chi-square test (α = 0.1) to determine the heterogeneity. When *I*^2^ ≤ 50%, it is considered acceptable. When *I*^2^ > 50%, subgroup analysis should be performed to identify potential causes and record them.

### 4.8 Data synthesis and grading of quality of evidence

RevMan 5 (version 5.3, Cochrane, London, UK) was used for statistical analysis of data. Risk ratios were used for binary variables and mean difference was used for continuous variables. Heterogeneity analysis will be conducted by heterogeneity test, *P* and *I*^2^ represent the size of the heterogeneity among multiple studies. When *P* is greater than .1 and *I*^2^ is less than 50%, it suggests heterogeneity is small, on the contrary, it suggests heterogeneity is large. Heterogeneity is mainly handled by subgroup analysis. Sensitivity analysis is used to test the reliability of the overall effect.

### 4.9 Subgroup analysis

When the heterogeneity test results are heterogeneous, we will conduct subgroup analysis to explore the possible causes of heterogeneity. The effects of different types of acupuncture therapy including design scheme, severity of illness, age, sex, and mild or severe AP were analyzed. We will also delete low-quality and/or medium-quality studies to check the robustness of the results.

### 4.10 Sensitivity analysis

Sensitivity analysis will be used to test the quality of the research contained in the sampled documents. The stability of the conclusions can be tested by reanalyzing the conclusions by inputting missing data and changing the type of research.

### 4.11 Ethics and dissemination

The results of the system review will be published in peer-reviewed journals, disseminated at relevant meetings, or disseminated in peer-reviewed publications, and we use aggregated published data to exclude individual patient data, so ethical approval, and informed consent are not required.

## 5 Discussion

For different AP conditions, previous studies provided some evidence on acupuncture's clinical effect on relieving abdominal pain or distension, including early stage AP complicated with intestinal paralysis, severe acute pancreatitis (SAP) with paralytic ileus, and septic gastrointestinal dysfunction, which might be a severe complication of AP.^[10,11,14–17]^

Moreover, mechanism studies found that acupuncture can reduce the inflammatory response in AP by regulating inflammatory factors. A study found that electroacupuncture may reduce the severity of AP by inducing anti-inflammatory effects, elevates serum concentrations of interleukin (IL)-10 , and decreases the C-reactive protein level in AP patients; meanwhile, electroacupuncture also reduced the time to refeeding.^[18]^ Another study found that electroacupuncture decreased tumor necrosis factor-α and increased IL-10 value in SAP patients, and relieved their associated lung injuries.^[19]^ An animal study figured that acupuncture at ST25 acupoint might have a therapeutic effect on SAP model through inhibition of nuclear factor-κB expression and a reduction in the release of proinflammatory cytokines.^[20]^ Another experiment found that electroacupuncture at the ST36 acupoint reduced the serum levels of some cytokines, including tumor necrosis factor, monocyte chemotactic protein-1, IL-6, and interferon-γ.^[21]^ Therefore, in 2013, an expert consensus on the AP management in China recommended acupuncture.^[22]^

Besides the VAS scores and clinical data, including time of pain and distension relieve, time of first bowel sound, time of first defecation, hospitalization length, and the adverse events, the APACHE II is also selected as one of outcomes of our study; this scoring system has been recognized as the gold standard in intensive therapy for years. Published in 1985, APACHE II is used to calculate the risk of hospital death. It is a useful prognostic scoring system for predicting the AP severity, and can be of vital importance in determining the group of patients who have more chance of requiring tertiary care during the course of treatment.^[23,24]^

To describe the strength of evidence, this review will use the GRADE approach.^[25]^ The GRADE methodology includes the assessment of the quality of evidence, which includes the risk of bias, inconsistency, immediacy, precision, and effect size. The grade of the recommendation is the degree of certainty that the implementation of an intervention has more benefit than harm. The grade will be determined based on the effect size, level of evidence, and resources.

A recent SR and meta-analysis investigated acupuncture as an adjuvant treatment for AP, but without focusing on abdominal pain and distension relieving, and only few studies that reported abdominal pain and distension were included.^[26,27]^ Thus, for both clinical practice and further research, we hope our work will provide the newest evidence of acupuncture treating abdominal pain and distension for AP.

## Author contributions

**Conceptualization:** Xinyun Zhu, Xianglei Li, Fengya Zhu.

**Data curation:** Xinyun Zhu, Fengya Zhu, Yuemeng Zhao.

**Formal analysis:** Xinyun Zhu, Lijie Yang.

**Funding acquisition:** Jie Wu.

**Methodology:** Xianglei Li.

**Project administration:** Yueheng Xiong, Jie Wu.

**Software:** Zimeng Li, Yuemeng Zhao.

**Supervision:** Jie Wu.

**Validation:** Lijie Yang, Zimeng Li.

**Visualization:** Fengya Zhu, Yueheng Xiong.

**Writing – original draft:** Xinyun Zhu, Lijie Yang.

**Writing – review & editing:** Andrea Craemer, Ying Lan.
